# Pepsin-Soluble Collagen from the Skin of *Lophius litulo*: A Preliminary Study Evaluating Physicochemical, Antioxidant, and Wound Healing Properties

**DOI:** 10.3390/md17120708

**Published:** 2019-12-16

**Authors:** Wen Zhang, Jiawen Zheng, Xiaoxiao Tian, Yunping Tang, Guofang Ding, Zuisu Yang, Huoxi Jin

**Affiliations:** Zhejiang Provincial Engineering Technology Research Center of Marine Biomedical Products, School of Food and Pharmacy, Zhejiang Ocean University, Zhoushan 316022, China; zhangwen1225z@163.com (W.Z.); jwzheng1996@163.com (J.Z.); TIANXIAOXIAO0208@163.com (X.T.); tangyunping1985@zjou.edu.cn (Y.T.); dinggf2007@163.com (G.D.); abc1967@126.com (Z.Y.)

**Keywords:** *Lophius litulon* skin, pepsin-solubilized collagen, characterization, antioxidant activity, biocompatibility

## Abstract

The structure of pepsin-solubilized collagen (PSC) obtained from the skin of *Lophius litulon* was analyzed using the sodium dodecylsulphate polyacrylamide gel electrophoresis (SDS-PAGE), Fourier transform infrared spectroscopy (FTIR), and scanning electron microscopy (SEM). SDS-PAGE results showed that PSC from *Lophius litulon* skin was collagen type I and had collagen-specific α1, α2, β, and γ chains. FTIR results indicated that the infrared spectrum of PSC ranged from 400 to 4000 cm^−1^, with five main amide bands. SEM revealed the microstructure of PSC, which consisted of clear fibrous and porous structures. In vitro antioxidant studies demonstrated that PSC revealed the scavenging ability for 2,2-diphenyl-1-picrylhydrazyl (DPPH), HO·, O_2_^−^·, and ABTS·. Moreover, animal experiments were conducted to evaluate the biocompatibility of PSC. The collagen sponge group showed a good biocompatibility in the skin wound model and may play a positive role in the progression of the healing process. The cumulative results suggest that collagen from the skin of *Lophius litulon* has potential applications in wound healing due to its good biocompatibility.

## 1. Introduction

Collagen, a biological macromolecule is one of the most abundant proteins in both invertebrates and vertebrates [1]. Collagen is mainly found in fibrillar connective tissue and widely used in the food, cosmetic, and nutritional health industries. In addition, highly stable collagen fibers formed by cross-linking and self-aggregation can be used as biomaterials to prepare a variety of practical scaffolds [2]. During wound healing, collagen initiates a signaling cascade that produces matrix metalloproteinases by activating integrin, which degrades the collagen matrix and causes keratinocytes to migrate [3]. The degradation products of collagen can be absorbed by cells and have antioxidation and immunomodulation biological effects [3,4]. Due to their excellent biocompatibility, antioxidant abilities, low immunogenicity, and extensive sources, collagen has become a research hotspot and main target of ideal biocompatibility carrier. For example, Itoh et al. studied the biocompatibility, bone conductivity and effectiveness of the new hydroxyapatite/type I collagen (HAp/Col) composite as a carrier of recombinant human bone morphogenesis protein. The results supported the idea that HAp/Col has high bone conduction activity and can induce bone remodeling units [5]. The study of Lukasiewicz et al. suggested that the collagen coating extracted by acetic acid greatly reduced the visceral adherence to the polypropylene mesh and did not increase complications or cause changes in the binding of the polypropylene mesh [6]. Chen et al. studied the antioxidant activity of ASC and PSC obtained from *Nibea japonica* swim bladders, and pointed out that PSC has potential application value in wound healing [7]. 

In general, ROS and antioxidants are balanced in healthy individuals. However, this balance can be disorganized in a wound [8]. Increased production of ROS due to injury and oxidative stress may result in poor wound healing [9]. Therefore, it is essential for wound healing to maintain the balance of ROS. Shao et al. studied the lipid peroxidation rate of diabetic rats using Plumbagin, and the results indicated that Plumbagin significantly accelerated wound healing by increasing the levels of antioxidant enzymes such as SOD, CAT, GPx, GR, and GST in diabetic rats [10]. The study of Abood et al. revealed that external use of P. macrocarpa fruit extract can accelerate wound healing and reduce tissue injury by increasing the activity of SOD, CAT, and MDA antioxidant enzymes [11].

*Lophius litulon* belongs to the Lophiidae and is a kind of deep-sea fish found in the Atlantic, Pacific, and Indian oceans. It is a commercially valuable aquatic resource, and fish fillets, stomachs, intestines, and livers are considered a delicacy in Japan, Korea, and China [12]. However, during processing, *Lophius litulon* skin is often discarded as a by-product, resulting in environmental pollution and waste. Chi et al. isolated three pentapeptides from muscle protein hydrolysates in monkfish (*Lophius litulon*) and evaluated their antioxidant activity [13]. Ma et al. showed that collagen peptides extracted from *Lophius litulon* skin had good antioxidant activity in vitro [14]. However, the physicochemical and biocompatibility properties of collagen extracted from *Lophius litulon* skin have not been reported. Therefore, in this study, we conducted a preliminary study evaluating physicochemical, antioxidant and biocompatibility properties of pepsin-solubilized collagen (PSC) from the skin of *Lophius litulon*. 

## 2. Results and Discussion

### 2.1. Sodium Dodecylsulphate Polyacrylamide Gel Electrophoresis (SDS-PAGE) Analysis

**Figure 1:**
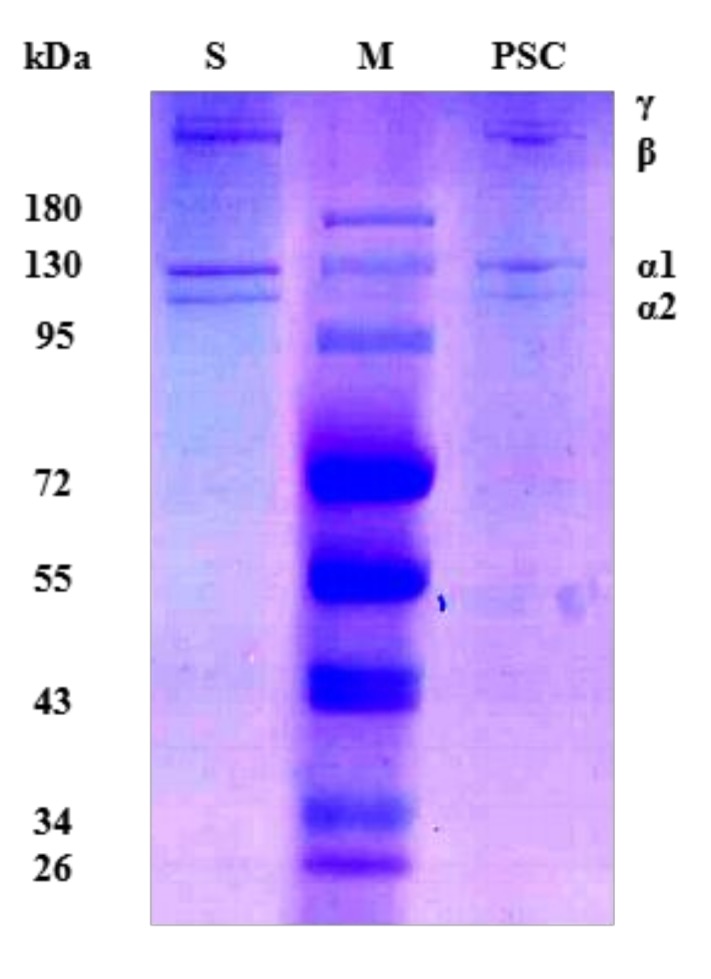
Sodium dodecyl sulfate-polyacrylamide gel electrophoresis (SDS-PAGE) analysis of pepsin-solubilized collagen (PSC) from *Lophius litulon* skin. M: Prestained color protein markers; S: Collagen type I from bovine Achilles tendon was used as standard.

It can be seen from the SDS-PAGE diagram of PSC (Figure 1) that the extracted components have four peptide chains: γ, β, α1, and α2. There are at least two different α chains (α1 and α2) in collagen type I [15]. It can be seen that the α1 chain is approximately twice as dense as α2, which is in accordance with the characteristics of collagen type I [16]. Furthermore, the PSC from *Lophius litulon* skin is similar to other marine fish source collagens, such as collagens from bigeye tuna skin and *Nibea japonica* skin [16,17].

### 2.2. Amino Acid Composition

We determined the amino acid composition of pepsin-solubilized collagen (PSC) from the skin of *Lophius litulon* and the results were used g/100 g as expression. As shown in Table 1, the collagen from Lophius litulon skin was rich in glycine (Gly), proline (Pro), glutamicacid (Glu), and alanine (Ala). Generally, the content of Gly in collagen was the highest, and the proportion of Gly in the total amino acid components of PSC was 23.85%. The content of hydroxyproline (Hyp) in PSC from the skin of *Lophius litulon* was 1.554 g/100 g, which was close to the content of Hyp in other collagen [18]. Collagen which is rich in hydrophobic amino acids has been shown to demonstrate high antioxidant properties [19,20]. Mcgavin et al. purified the 72 kD protein on receptor(s) for bone sialoprotein (BSP) in Staphylococcus aureus cells, and their amino acid composition analysis showed that the 72 kD protein contained 28.0% hydrophobic amino acids in total [21]. The content of hydrophobic amino acids in the PSC accounted for 22.16% in total, indicating that the collagen has potential antioxidant properties.

### 2.3. Relative Solubility

**Figure 2:**
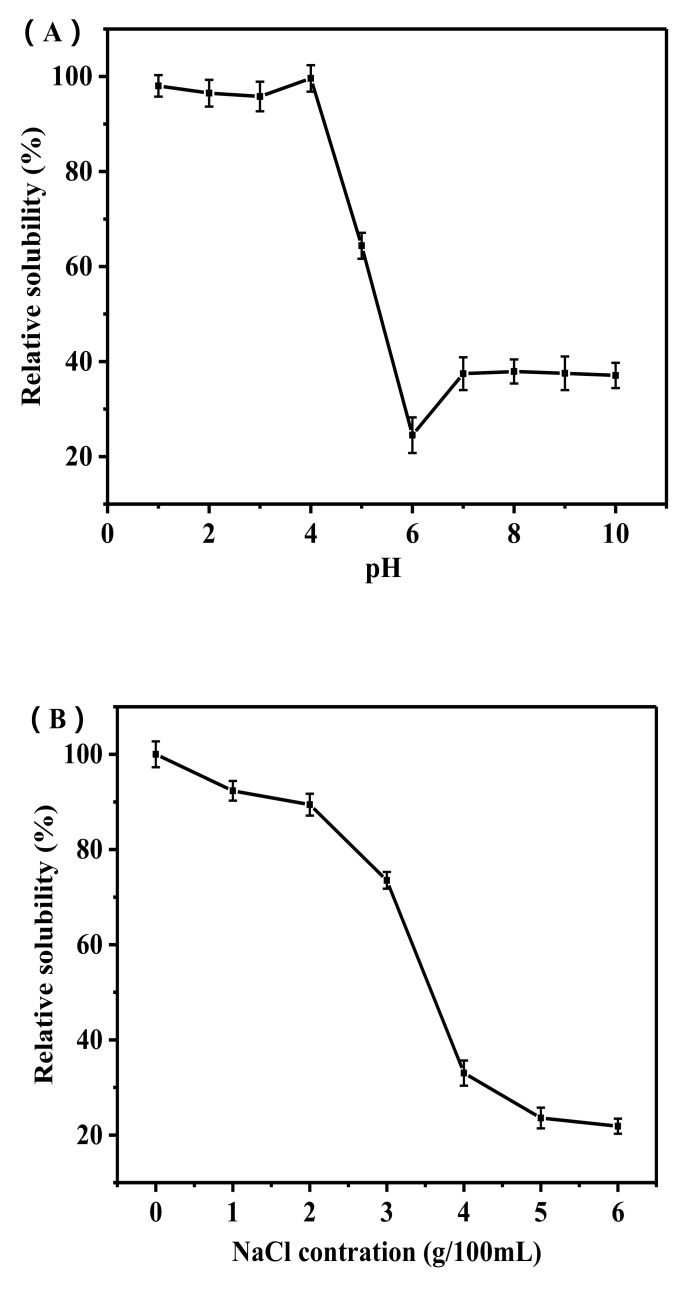
Effect of pH (**A**) and NaCl concentration (**B**) on the solubility of PSC.

The solubility of the PSC against pH is shown in Figure 2A. PSC had good solubility in the range of 1 to 4 of pH. When the pH was greater than 4, the solubility of PSC decreased sharply with rising pH. Low solubility of PSC was caught sight of an alkaline pH from 8 to 10. Generally, the solubility of collagen is the lowest near its iso-ionic point [22,23]. Our results indicate that the iso-ionic point of PSC may be approximately pH 6.

Increases in NaCl concentration increase ion strength and enhance hydrophobic interaction between protein chains, thus reducing the solubility of collagen and leading to protein precipitation [24]. Since the average salt concentration of the ocean is 3.5%, the salt concentrations in this study ranged from 0% to 6%. Figure 2B displays changes in collagen solubility with changing NaCl concentration. Small increments of NaCl concentration (≤2 g/100 mL) seemed to have a moderate impact on the collagen solution, leading to a slight decrease in relative solubility (100%–90%). However, solubility was rapidly decreased when NaCl level were higher than 2 g/100 mL. The above results are consistent with PSC-SC from the cartilage of Siberian sturgeon and collagens from the skin of pufferfish [25,26].

### 2.4. Fourier Transform Infrared (FTIR) Spectroscopy

**Figure 3:**
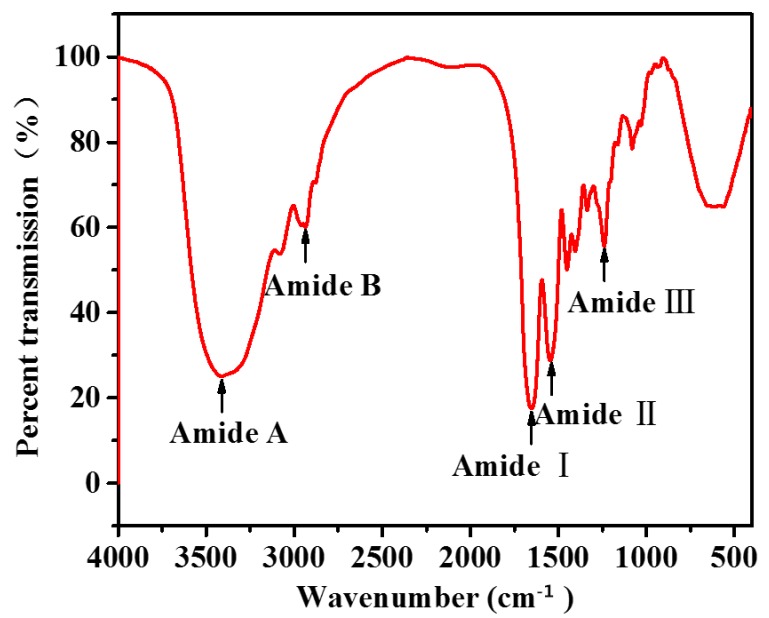
FTIR analysis of PSC from *Lophius litulon* skin.

Figure 3 presents the characteristic peaks conforming with the main absorption bands in the FTIR spectra for PSC. The FTIR spectra for PSC ranged between 400 and 4000 cm^−1^. These peaks corresponded to five major amide bands, including amide A, amide B, amide I, amide II, and amide III, which were evident in the amino acids’ composition and high ratios of proline and hydroxyproline in the collagen molecule. The amide A bands of PSC were measured at a wavelength of 3408.14 cm^−1^. Amide A bands are commonly associated with a free N–H stretching vibration and shows the presence of hydrogen bands, which usually appear in the range of 3400–3440 cm^−1^ [27]. The amide B bands of PSC were measured at a wavelength of 2959.48 cm^−1^, consistent with the asymmetric extension of CH_2_ [25]. The amide I bands of PSC were measured at 1653.66 cm^−1^, with a characteristic absorption wavelength within 1600–1700 cm^−1^, which is associated with the stretching vibration of the carbonyl group [28]. The amide II bands of PSC were measured at a wavelengths between 1550–1600 cm^−1^, which can be owing to a N–H bending vibration coupled with a C–N stretching vibration [29]. Futhermore, the amide III bands (1220–1320 cm^−1^) were measured at a wavelength of 1239.84 cm^−1^, which may be affected by N–H deformation and C–N stretching vibration [30]. The FTIR spectra of PSC has significant collagen characteristics and is similar to the skin of *Nibea japonica* [31] and the scale of *Oreochromis niloticas* [32].

### 2.5. Scanning Electron Microscope (SEM) Analysis

**Figure 4:**
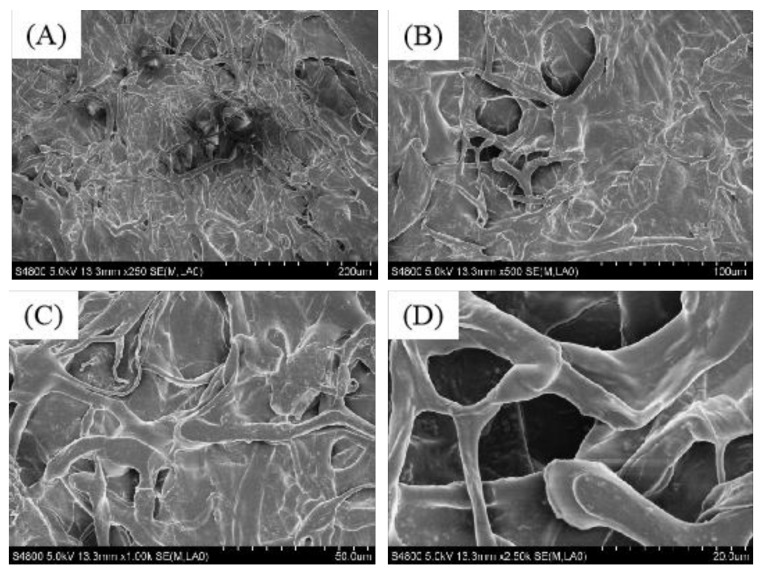
SEM images of PSC from *Lophius litulon* skin. (**A**) ×250, (**B**) ×500, (**C**) ×1000, and (**D**) ×2500.

In recent years, collagen has proven to be of great value in the field of biomedicine and for various medical applications. Therefore, it is important to understand the surface morphology and micromorphology of collagen. The surface area and ultrastructure of the PSC was observed using SEM microstructure to evaluate its potential applications. SEM images of PSC taken at ×250, ×500, ×1000, and ×2500 are presented in Figure 4. The SEM images of PSC displayed a complex, multilayered, polymeric fibrous meshwork appearance. At ×500 magnification, the fibrous and porous structures were clearly visible. PSC with inter-connected porous structures are similar to other marine collagens, such as collagen from the skin of sole fish and Pacific cod (*Gadus macrocephalus*) [33,34]. The microstructure of PSC indicates that it has great potential for application as a wound excipient or drug carrier in biomedical engineering and medicine.

### 2.6. Antioxidant Activity

One of the major factors that plays an important part in wound healing is the regulation and oxidation of inflammation. Oxidative stress plays a crucial role in the progression of wound healing [35]. Reactive oxygen species (ROS) are a vital component of oxidative stress. Therefore, the existance of antioxidants is considered to be an essential factor in successful wound healing [36,37]. Besides, a large number of experiments have demonstrated that marine collagen and collagen peptides exhibit antioxidant effects. In this study, we assessed the antioxidant properties of collagen extracted from the skin of *Lophius litulon* using four free radicals: DPPH·, ABTS·, HO·, and O_2_-·.

**Figure 5:**
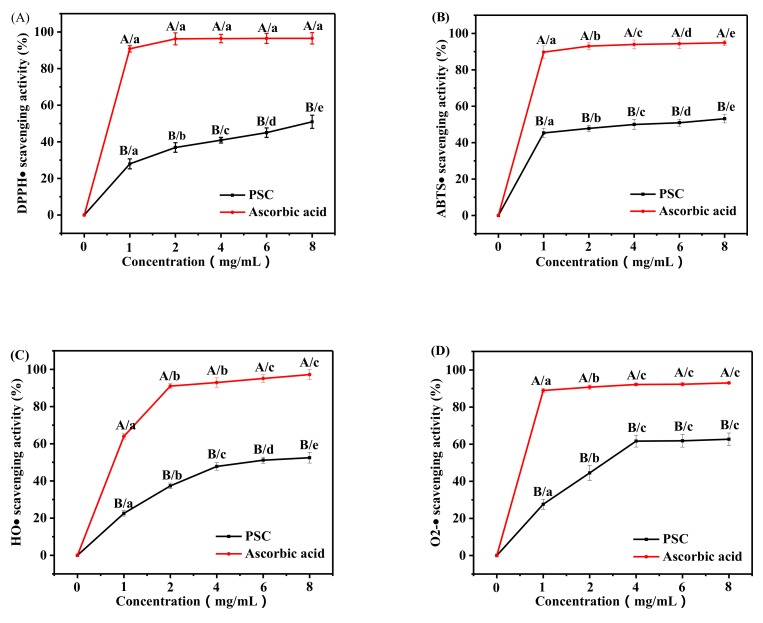
(**A**) 2,2-diphenyl-1-picrylhydrazyl (DPPH)·, (**B**) ABTS·, (**C**) HO·, and (**D**) O_2_-· scavenging activities of PSC from *Lophius litulon* skin. (A–B) Values with different letters indicated significant differences in different samples at the same concentrations (*p* < 0.05). (a–e) Values with different letters indicated significant differences in the same samples at the different concentrations (*p* < 0.05).

As shown in Figure 5, PSC was able to scavenge DPPH·, ABTS·, HO·, and O_2_-·, but its scavenging ability for these four free radicals was lower than those of the positive control (ascorbic acid). Additionally, the scavenging activities of collagen on free radicals was dose dependent. When the concentration of collagen was high, the scavenging activities of free radicals tended to be stable. These phenomena were similar to other previously examined marine collagens. In addition, compared to some other marine collagens, the scavenging activities of free radicals by collagen from *Lophius litulon* skin have certain advantages. For example, the scavenging activities of DPPH·, HO·, and O_2_-· by collagen peptides from tilapia skin were lower than those of PSC extracted from *Lophius litulon* skin at the same concentration [38]. The concentrations of ASC and PSC from *Nibea japonica* swim bladders were higher than that of PSC from *Lophius litulon* skin when the clearance rate of ABTS· reached 50%. Moreover, the scavenging activities of both HO· and O_2_-· by ASC and PSC from *Nibea japonica* swim bladders did not reach 50% even at concentrations of 10 mg/mL, which were obviously lower than that of PSC from *Lophius litulon* skin [7]. These results indicated that the PSC from *Lophius litulon* skin has a moderate antioxidant capacity.

### 2.7. Estimation of Antioxidant Status in Animal Serum

Malondialdehyde (MDA) is widely used for indicator of lipid peroxidation, and an increase in MDA content suggests lipid peroxidation damage during skin aging [39]. Superoxide dismutase (SOD) is present in every cell of the human body and has been shown to protect cells and tissues against oxidative stress [38]. SOD reduces superoxide anion radicals to generate hydrogen peroxide, which can then be disintegrated into water and oxygen by glutathione peroxidase (GSH-Px) and catalase (CAT), and preventing the formation of hydroxyl radicals [38,40].

**Figure 6:**
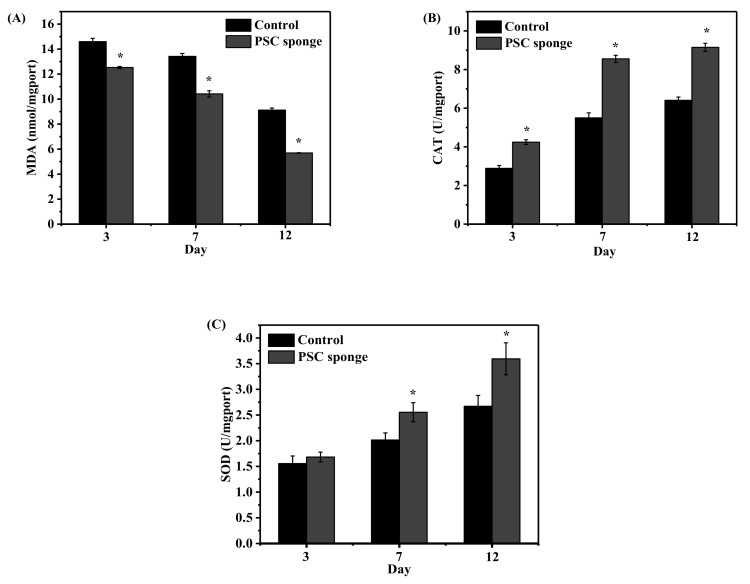
Effects of PSC on serum antioxidant activity in mice. (**A**) MDA, (**B**) CAT, and (**C**) SOD. Data are expressed as the mean ± SD (*n* = 5). * a significant difference when compared to the control (*p* < 0.05).

As shown in Figure 6A, MDA content gradually decreased as the number of days increased, and the content of the PSC sponge group was always lower than that of the control group. There were significant differences in MDA content between the PSC sponge group and the control group at 3, 7, and 12 days (*p* < 0.05). Figure 6B and 6C show that the content of CAT and SOD in the serum of mice gradually increased over time. Moreover, the content of the PSC sponge group was always higher than that of the control group. In addition, compared with the control group, CAT content in the PSC sponge group was significantly different at 3, 7, and 12 days, and SOD content in the PSC sponge group had a significant difference at days 7 and 12 days (*p* < 0.05). The above results demonstrate that PSC slowed the formation of MDA andenhanced the activity of CAT and SOD.

### 2.8. Evaluation of Biocompatibility in the Skin Wound Model

**Figure 7:**
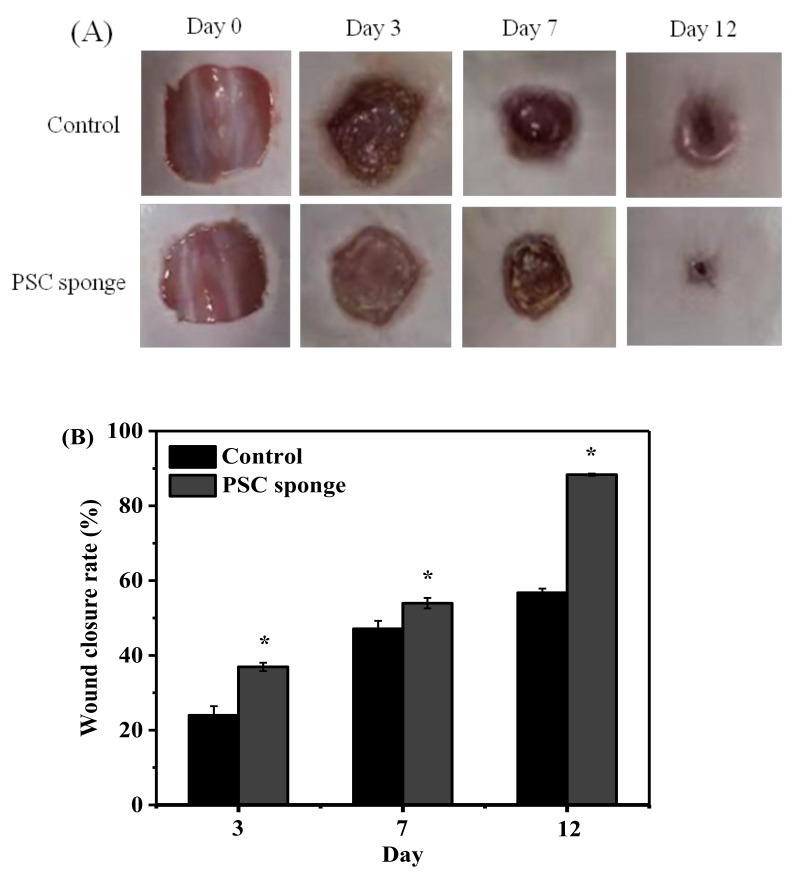
Effect of PSC in wound healing. (**A**) Representative photographs on days 0, 3, 7, and 12. (**B**) Wound contraction (%) on days 3, 7, and 12. Data are expressed as the mean ± SD (*n* = 5). *, a significant difference when compared to the control (*p* < 0.05).

Many papers have been reported that the antioxidant defense can reduce ROS production and accelerate wound healing. It was shown that PSC from *Lophius litulon* skin has a certain antioxidant function based on its ability to scavenge free radicals and increase the content of SOD and CAT in the serum of mice. Therefore, the PSC from *Lophius litulon* skin may have potential applications in wound healing. Of course, another prerequisite for application in the wound healing purposes is that the PSC must have good biocompatibility. In this study, a skin wound model was preliminary tested for evaluation of biocompatibility of PSC and its potential for wound healing. Photographs were taken on days 0, 3, 7, and 12 using a digital camera to assess wound status. As presented in Figure 7A, PSC sponge treatment did not significantly cause epithelial necrosis and wound deterioration, indicating that PSC sponge has no significant toxic effect on skin cells and negative stimulating effect on wounds. Furthermore, the wound healing rates were 23.99%, 47.12%, and 56.77% in the control group, respectively, on days 3, 7, and 12, while the wound healing rates were 36.92%, 53.97%, and 88.34% in the PSC sponge group on days 3, 7, and 12 (Figure 7B).

To evaluate the progression of the healing process as a proof that the sponge is biocompatible, histological studies were performed on the wound tissue at 3, 7, and 12 days.

**Figure 8:**
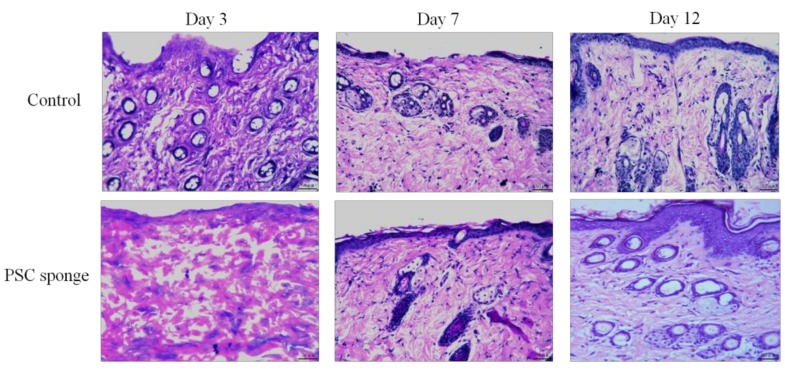
Histopathological observation of wound tissue of skin of the control and PSC sponge groups with H&E staining on days 3, 7, and12 (magnification: ×200).

As shown in Figure 8, after 3 days the thickness of epidermal cells in the control group was smaller and the epidermis was partially exfoliated. In addition, a large number of inflammatory cells infiltrated the dermis. Granulation tissue and neovascularization, fibroblast proliferation, and collagen fiber accumulation were virtually absent in the control group. There was a thinner layer of epidermal cells in the PSC sponge group, and some inflammatory cells still existed in the dermis. There was also some granulation tissue formation in the collagen sponge group.

At day 7, 1 to 3 layers of epidermal cells could be observed in the control group, and some areas of epidermal cells had not yet grown. A large number of inflammatory cells were still infiltrated into the dermis. At this time, we observed new granulation tissue and blood vessels, fibroblast proliferation and collagen fiber accumulation. The epidermal cells of PSC sponge treated tissues reached 3–5 layers, and had fewer inflammatory cells. Sebaceous glands and hair follicle tissue increased, fibroblasts proliferated, and collagen fibers accumulated to a greater extent than at day 3.

By day 12, the epidermal cells in the control group had 3-4 layers, and the dermal cells were infiltrated with a large number of inflammatory cells. At this point, the new granulation tissue was thickened, new angiogenesis increased, and sebaceous cells and hair follicle tissue began to form. Meanwhile, in the PSC sponge group, there were 5–8 layers of epidermal cells were observed, and inflammatory cells in the dermis were almost non-existent. The collagen sponge group demonstrated higher epidermal regeneration, granulation tissue regeneration, angiogenesis, fibroblast proliferation, and collagen fiber deposition.

It was seen from the results that PSC sponge treatment could promote the epidermal cell proliferation and reduce the production of inflammatory cells in the skin wound model. The regeneration of epithelial cells, the reduction in the number of inflammatory cells, the augment in the thickness of granulation tissue, and the deposition of collagen fibers are considered to be signs of progress in the healing process [41]. Therefore, our results indicated that the PSC sponge from the skin of *Lophius litulon* has good biocompatibility and the potential for wound healing.

## 3. Materials and Methods

### 3.1. Materials and Chemical Reagents

The pepsin-soluble collagen (PSC) used in this experiment was obtained by laboratory extraction and separation. The prestained color protein marker (cat. no. P0068) was obtained from Beyotime Biotechnology (Shanghai, China). Collagen type I (cat. no. C8060) was provided by Solarbio (Beijing, China). MDA, CAT, SOD and Hematoxylin-eosin (H&E) staining kits were supplied by the Nanjing Jiancheng Bioengineering Institute (Nanjing, China). The enzyme-linked immunosorbent assay (ELISA) kits for detecting IL-6, IL-1β, and tumor necrosis factor (TNF-α) were purchased from Elabscience Biotechnology Co., Ltd. (Wuhan, China).

### 3.2. SDS-PAGE Analysis

According to the method described by Tang et al. [16], PSC was analyzed using SDS-PAGE. PSC samples were first dissolved in 0.5 M acetic acid and mixed with 5× loading buffer. The mixture was boiled at 100 °C for 5 min and placed in a centrifuge (12,000 rpm, 5 min) to remove the remaining fragments. Electrophoresis was performed using 7.5% gel to estimate the molecular weight of PSC. Collagen type I was used as a positive control.

### 3.3. Amino Acid Analysis

The amino acid composition of the PSC samples were hydrolyzed using 6 M HCl at 110 °C for 24 h and then vaporized. The hydrolyzates were neutralized in a 25 mL citric acid buffer and analyzed using an amino acid analyzer (L-8800, Hitachi, Tokyo, Japan).

### 3.4. Relative Solubility

The lyophilized PSC sample (3 mg/mL) was dissolved in 0.5 M acetic acid. The PSC solution (8 mL) was then transferred to a 50 mL centrifuge tube and the pH of PSC solution was adjusted using either 6 M HCl or 6 M NaOH to a final pH of 1.0 to 10.0, and was maintained at 10 mL through deionized water. The mixture was centrifuged at 4 °C at 12,000 rpm for 10 min and the protein content in the supernatant was measured using the BCA kit from the Nanjing Jiancheng Bioengineering Institute.

The influence of NaCl on PSC solubility was determined as follows: the PSC (3 mg/mL) was dissolved in 0.5 M acetic acid, and 5 mL NaCl (in 0.5 M acetic acid) in different solution concentrations (0%, 2%, 4%, 6%, 8%, 10%, and 12%) were added to the solution until the final concentration was either 0%, 1%, 2%, 3%, 4%, 5%, or 6%. The mixed solutions were then stirred for 30 min at 4 °C and then centrifuged (4 °C, 12,000 rpm, 10 min). The protein content in the supernatant was measured as described above. Relative solubility was calculated as the ratio of the protein concentration at the current pH to the protein concentration at the maximum pH.

### 3.5. FTIR Spectroscopic Analysis

The FTIR spectra of lyophilized PSC samples were received using a FTIR spectrometer (Bruker, Rheinstetten, Germany). The infrared spectra were recorded at a resolution of 1 cm^−1^ and a wavelength range of 4000–400 cm^−1^. Analysis of the spectral data were analyzed using ORIGIN 8.0 software (Thermo Nicolet, Madison, WI, USA).

### 3.6. SEM Analysis

The collagen samples were mounted on a blade with two-sided adhesive tape. They were then placed inside a sputter for gold sputtering and the images of sputtered specimens were observed using a scanning microscope (JSM-840, JEOL, Tokyo, Japan) at ×250, ×500, ×1000, and ×2500 magnification.

### 3.7. Antioxidant Activity

The scavenging activity of PSC for DPPH·, ABTS·, HO·, and O_2_- radicals was measured according to the procedures described by Chen et al. [7], with ascorbic acid used as a positive control.

#### 3.7.1. DPPH Radical Scavenging Activity

One milliliter of samples containing different concentrations (1–8 mg/mL) were added to different centrifuge tubes (5 mL), and then 250 μL DPPH (0.02%) ethanol and 1.0 mL absolute ethanol were added. The mixture was left in the dark for 30 min and the absorbance (As) was measured at 517 nm. The sample replaced with deionized water was used as the control group (Ac), and the DPPH replaced with ethanol was used as the blank group (Ab). The calculation formula of DPPH· scavenging activity is as follows:

$$\mathrm{DPPH}\cdot \mathrm{scavenging}\;\mathrm{activity}\;\%=\frac{\left[1-\left(\mathrm{As}-\mathrm{Ab}\right)\right]}{\mathrm{Ac}}\times 100\%$$

#### 3.7.2. ABTS Radical Scavenging Activity

One milliliter of ABTS radical diluent and 1 mL samples with different concentrations (1–8 mg/mL) were added into different centrifuge tubes (5 mL). The mixture was left in the dark for 10 min and the absorbance was measured at 734 nm (As). Samples replaced with deionized water were used as the control group (Ac). The calculation formula of ABTS· scavenging activity is as follows:

$$\mathrm{ABTS}\cdot \;\mathrm{scavenging}\;\mathrm{activity}\;\left(\%\right)=\frac{\mathrm{Ac}-\mathrm{As}}{\mathrm{Ac}}\times 100\%$$

#### 3.7.3. Hydroxyl Radical Scavenging Activity

One milliliter of o-Phenanthroline solution (1.5 mM) was added in different centrifuge tubes (5 mL), and then 1mL samples of different concentrations (1–8 mg/mL) and the 1mL FeSO4 solution (1.5 mM) were added, respectively. Finally, 1.0 mL of 0.03% H_2_O_2_ solution was added to induced the reaction. The mixture was bathed in water at 37 °C for 90 min and its absorbance was measured at 536 nm (As). Samples replaced with deionized water were used as the control group (Ac), and the 0.03% H2O2 solution replaced with deionized water was used as the blank group (Ab). The calculation formula of HO· scavenging activity is as follows:

$$\mathrm{HO}\cdot \;\mathrm{scavenging}\;\mathrm{activity}\;\left(\%\right)=\frac{\left[1-\left(\mathrm{As}-\mathrm{Ab}\right)\right]}{\mathrm{Ac}}\times 100\%$$

#### 3.7.4. Superoxide Anion Radical Scavenging Activity

One-milliliter samples of different concentrations (1–8 mg/mL) were added into different centrifuge tubes (5 mL), and then 1 mL Nitro tetrazolium blue chloride (NBT) solution (2.52 mM) and 1 mL NADH (624 μM) was added. At last, 1 mL phenazine methosulfate (PMS) solution (120 μM) was added to induced the reaction. The mixture was bathed in water at 25 °C for 5 min and the absorbance was measured at 560 nm (As). Samples replaced with deionized water were set as control group (Ac). The calculation formula of O_2_-· scavenging activity is as follows:

$$O2-\cdot \;\mathrm{scavenging}\;\mathrm{activity}\;\left(\%\right)=\frac{\mathrm{As}-\mathrm{Ac}}{\mathrm{Ac}}\times 100\%$$

### 3.8. Animals Grouping and Wound Creation

Thirty male ICR mice (22–24 g) were provided by the Experiment Animal Center of Zhejiang Province (certificate no. SCXK 2014-0001). All the mice were kept under conventional and uniform conditions at 22 °C. After the mice were given 7 days to adapt to their new environment, they were randomly divided into two groups: the control group and the PSC sponge group. All mice were fed with the SPF grade mouse feed at 4 g/day.

The mice were narcosised with 4% chloral hydrate before undergoing the surgical procedure to create the wound. The hair was then removed from the mice and sterile surgical scissors were used to create a 1 cm diameter wound area. The control group was only treated with 0.9% saline on the wound area. In the PSC sponge group, the wound area was completely covered with a PSC sponge, and the PSC sponge was sterilized using ultraviolet radiation before use. Photographs were taken on days 0, 3, 7, and 12 using a digital camera to assess wound healing status, and Image J software was used to calculate wound area. The formula for wound closure rate (%) was as follows:

$$\mathrm{Wound}\;\mathrm{closure}\;\mathrm{rate}\;\left(\%\right)=\frac{0\;\mathrm{day}\;\mathrm{wound}\;\mathrm{area}\;-\;\mathrm{wound}\;\mathrm{area}\;\mathrm{on}\;a\;\mathrm{particular}\;\mathrm{day}}{0\;\mathrm{day}\;\mathrm{wound}\;\mathrm{area}}\times 100\%$$

On days 3, 7, and 12, five mice in each group were killed. Serum was collected to assess antioxidant levels and the wound area was carefully removed from each mouse. Each wound area was divided into two parts. One part was used for histopathological observation and the other was stored at −80 °C for analysis of inflammatory factor levels.

### 3.9. Assessment of Serum Antioxidant Levels

Blood was sampled from the eyes of the mice before each group of mice was killed. Serum and plasma were separated in a refrigerated centrifuge (4 °C, 5000 rpm, 5 min). The contents of MAD, CAT and SOD in the serum were measured using mouse MAD, CAT and SOD kits.

### 3.10. Histomorphological Observation

After collecting the wound tissue, the tissue was fixed in 4% paraformaldehyde solution for 24–48 h, then embedded in paraffin, cut into sections that were 4 microns in size, stained with a H&E staining kit, and sealed with a neutral gel. The histomorphological changes of the wound tissues in each group were observed under an optical microscope (CX31, Olympus) and photographed using a CCD-NC 6051 photographic system.

### 3.11. Statistical Analysis

The experimental data were analyzed and processed using IBM SPSS 19.0 statistical software (Armonk, NY, USA). All experiments were repeated in triplicate and the figures were expressed as mean ± standard deviation (SD). The data were analyzed using a one-way analysis of variance (ANOVA) test, and *p* < 0.05 values were considered to be statistically significant.

## 4. Conclusions

We studied the physicochemical properties, antioxidant activity, and biocompatibility of pepsin-solubilized collagen (PSC) obtained from the skin of *Lophius litulon*. The results of FTIR and SEM revealed that PSC had a three-step spiral structure and porous fiber network microstructure. PSC can scavenge the free radicals in a dose-dependent manner and increase the levels of SOD and CAT, suggesting that the PSC from *Lophius litulon* skin showed antioxidant activity. Furthermore, the antioxidant activity of PSC was higher than that of collagen peptides from tilapia skin and collagens from *Nibea japonica* swim bladders [7,38]. Excessive reactive oxygen species (ROS) can lead to a variety of chronic health problems, and increased antioxidant defense can reduce ROS production and accelerate wound healing [42]. At present, considerable attention has been paid to the use of fish-derived collagen and peptides for wound healing. Hu et al. reported that collagen peptides from the skin of Nile tilapia (Oreochromis niloticus) could enhance the process of wound healing [17]. The present finding suggested that the PSC from *Lophius litulon* skin has good biocompatibility and may be used as a biomaterial for wound healing in clinical and cosmetic fields. In the future, we will further explore the effect of PSC on wound healing.

## Figures and Tables

**Table 1:** Amino acid compositions of PSC from *Lophius litulon* skin.

Amino Acids	Content (g/100 g)
Aspartic acid (Asp)	2.529
Threonine (Thr)	1.310
Serine (Ser)	2.522
Glutamic (Glu)	4.708
Priline (Pro)	4.720
Glycine (Gly)	9.684
Alanine (Ala) *	4.009
Valine (Val) *	1.640
Methionine (Met) *	1.044
Isoleucine (Ile)	0.262
Leucine (Leu) *	1.312
Tyrosine (Tyr) *	0.000
Phenylalanine (Phe) *	0.991
Lysine (Lys)	1.462
Histidine (His)	0.931
Arginine (Arg)	3.484
Hydroxyproline (Hyp)	1.554

* represents hydrophobic amino acids.
